# Bacterial meningitis in Niger: an analysis of national surveillance data, 2003-2015

**DOI:** 10.11604/pamj.2018.30.235.15937

**Published:** 2018-07-30

**Authors:** Lawaly Maman Manzo, Sani Ousmane, Dan Dano Ibrahim, Maman Zaneidou, Jean Testa, Halima Boubacar Maïnassara

**Affiliations:** 1 Centre de Recherche Médicale et Sanitaire (CERMES), BP 10887, 634 Bd de la Nation, YNo34 Niamey, Niger; 2 Direction de la Surveillance et Riposte aux Epidémies (DSRE), Ministère de la Santé Publique, Niamey, Niger

**Keywords:** Niger, epidemiology, bacterial meningitis, surveillance

## Abstract

**Introduction:**

Bacterial meningitis (BM) is one of the most severe infectious disease in Niger republic. To best describe the trends of BM disease, meningitis surveillance data from the Centre de Recherche Medicale et Sanitaire (CERMES) and the Direction of Surveillance and Response to Epidemics (DSRE) were reviewed and analyzed.

**Methods:**

Data on number of notified cases of BM and on pathogens were analyzed during 2003-2015. Excel 2013 was used for trend analysis on the etiology of BM prevalence and incidence.

**Results:**

A total of 10051 cerebrospinal fluid (CSF) samples collected were confirmed by laboratory methods. The main etiologies of meningitis detected were *N. meningitidis* (82.1%), *S. pneumonia* (12.1%) and *H. influenza* (3.4%). *N. meningitidis* mostly affected children in the age groups of 5-9 years (32.9%) and 10-14 years (24.9%) with respective mean incidence of 14.9 and 11.3. The percentage estimate of *N. meningitidis* serogroup A (NmA) meningitis fell to 0% in 2015 while during the same year that of *N. meningitidis* serogroup C (NmC) and *N. meningitidis* serogroup W (NmW) reached 82.9% and 17% respectively.

**Conclusion:**

Overall, the epidemiological trends of the BM in Niger were dynamic. The emergence of NmC strains suggests that there may be an urgent need for serogroup C containing vaccines in Niger in the coming years.

## Introduction

Niger, a sub-Saharan country located in the center of the African “meningitis belt” that stretches from Senegal in the west to Ethiopia in the east is periodically experiencing important meningitis outbreaks. Mainly, three pathogenic microorganisms (*N. meningitidis, S. pneumoniae and H. influenza*) were the cause of more than 80% of bacterial meningitis in sub-Sahara Africa. These outbreaks, which could be highly aggressive in number of reported cases, were essentially due to NmA up to year 2000. In 2003, the World Health Organization and the Meningitis Vaccine Project, a joint WHO/PATH initiative supported Niger and other African countries to put in place an enhanced meningitis surveillance network [1]. The surveillance system is well organized with standard operating procedures for better record and management of data on suspected cases notified during an outbreak from peripheral health centers to the district medical surveillance, and further by broadcasting through radio, telephone, fax, or email to regional and national levels. Collected CSF specimen accompanied with a notification factsheet which includes personnel information of the patient and the relevant clinical information are sent to a national reference microbiology laboratory for meningitis located in CERMES for confirmation and further provides retrospective data on the epidemiology of epidemic BM in Niger over several year period. Several studies have been performed in CERMES and or through its collaborative centers evaluating the etiology of BM in Niger population. According to the significant burden associated with bacterial meningitis in Niger and throughout the world, a review of yearly reported data is necessary. Accurate determination of the etiology of BM by advanced molecular methods and estimating burden and length of an outbreak may significantly guide vaccine strategy. This study provides trends of BM outbreaks causing pathogens over 12 years.

## Methods

**Microbiological surveillance**: The microbiological surveillance program with characterization of infectious agents at national level is performed by CERMES with the direct collaboration of DSRE, Ministry of Public Health (MoPH) and the National Bureau of the World Health Organization (WHO) in Niger. CERMES complements the epidemiological surveillance of bacterial meningitis in Niger, its tendency, the burden of outbreaks with the identification and characterization of the etiology of bacterial meningitis notified in CERMES using a representative number of CSF samples collected throughout Niger [2].

**Collection of CSF and epidemiological data entry**: CERMES is involved in “passive” collection of CSF samples from sentinel hospital surveillance site where an EPID collection number is assigned and inscribed on the notification factsheet, then forwarded to CERMES. Collected CSF samples were either conserved in refrigerator or freezer in peripheral health center, or inoculated into Trans-Isolate medium referred as TI and sent for culture and identification in CERMES. Collected data through surveillance-patient factsheet and biological test results were inputted in database managed by MySQL.

**Laboratory methods**: Conventional bacteriological methods and molecular methods (such as PCR) [2] were used for the confirmation of bacterial meningitis from CSF specimens.

**Data analysis**: Epidemiological surveillance data from the Ministry of Public Health (MoPH), WHO-Africa regional office bulletins and CERMES were extracted and analyzed using Microsoft Excel 2013 (Microsoft Corporation). Data on notified cases were taken from DSRE/MoPH and those for pathogens were taken from CERMES and the WHO bulletins. Population estimates for 2003 to 2015 were obtained from the National Institut of Statistic (INS) and the DSRE.

## Results

**Figure 1:**
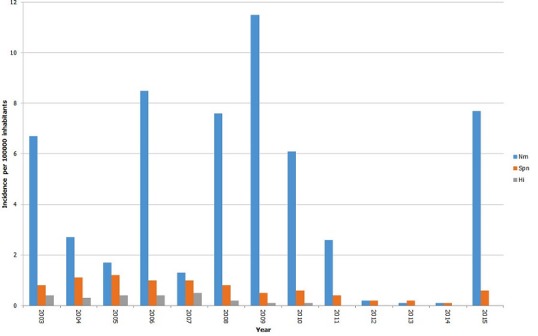
Annual incidences of bacterial meningitis per 100,000 population, Niger, 2003-2015

**Figure 2:**
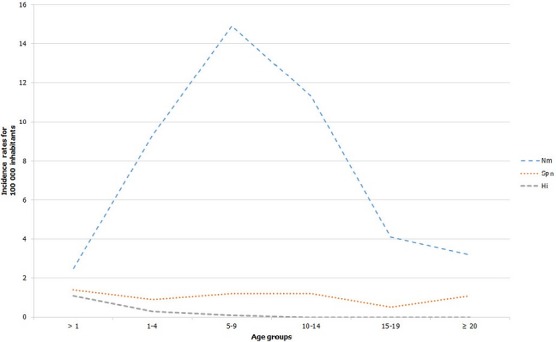
Incidence rates of main etiologies of bacterial meningitis by age group, Niger, January 2003 to December 2015

From 2003 to 2015, the MoPH of the republic of Niger reported 52.333 suspected cases of BM. The largest number of cases was observed in 2009 (n = 13,943). A progressive significant decline in the number of reported cases was observed from 2010 (n = 2,908), 2011 (n = 1,344), 2012 (n = 293), 2013 (n = 357) and 2014 (n = 303). The number of laboratory confirmed cases of meningitis was 10.051 (41.4%) with *N. meningitidis, S. pneumoniae* and *H. influenza* representing 82.1%, 12.1% and 3.4% of cases respectively. NmA was the main pathogen isolated during the period 2003-2009. From 2010 to 2013, NmW was the main serogroup of Nm responsible of BM before its replacement by NmC in 2015 which become the major pathogen (Table 1). *S. pneumoniae* represent 12.1% of all confirmed cases of meningitis throughout the years under review. Cases due to *H. influenza* were found more frequently in 2003-2010 than in the following years (4.1% of confirmed BM cases in 2003-2010 vs 0.9% in 2011-2015) (Table 1) [3,4]. The year incidence rates of the isolated strains are presented in (Figure 1). The incidence per 100,000 of Nm meningitis appeared considerably pronounced in 2003 (6.7), 2006 (8.5), 2008 (7.6), 2009 (11.5), 2010 (6.1) and 2015 (7.7), while the incidences of *S. pneumoniae* and *H. influenza* meningitis appeared relatively low (≤ 1.2) from 2003 to 2015. The average incidence of bacterial meningitis according to pathogen and age during the 12 epidemic years are shown in Figure 2. Nm has the highest incidence rates in children aged 5 to 14 years. The incidence rates of *S. pneumoniae*, was relatively constant within all age groups. *H. influenza* mostly affect children aged less than 5 years.

**Table 1:** Epidemiological and microbiological surveillance data of bacterial meningitis in Niger, 2003-2015

Year	Number of analyzed CSF	Spn	Hi	Nm (all types)	Nm A	Nm C	Nm W	Nm X	Nm Y
2003	996	91(9.1%)	48(4.8%)	787(79.0%)	710(90.2%)	0	65(8.3%)	2(0.30%)	4(0.5%)
2004	521	137(26.3%)	39(7.5%)	335(64.3%)	275(82.1%)	0	30(8.9%)	14(4.20%)	3(0.9%)
2005	409	152(37.2%)	44(10.8%)	207(50.6%)	134(64.7%)	0	20(9.7%)	42(20.30%)	0
2006	1,301	132(10.1%)	58(4.5%)	1,101(84.6%)	503(45.7%)	0	21(1.9%)	559(50.80%)	1(0.1%)
2007	388	134(34.5%)	62(16%)	172(44.3%)	151(87.8%)	0	5(3.3%)	9(5.20%)	0
2008	1,255	117(9.3%)	33(2.6%)	1,082(86.2%)	1,067(98.6%)	0	0	5(0.50%)	0
2009	1,852	78(4.2%)	12(0.6%)	1,696(91.6%)	1,654(97.5%)	0	11(0.6%)	15(0.90%)	1(0.6%)
2010	1,062	93(8.8%)	22(2.1%)	921(86.7%)	243(26.4%)	0	665(72.2%)	1(0.40%)	0
2011	494	70(14.2%)	2(0.4%)	410(83.0%)	5(1.2%)	0	402(98.0%)	1(0.20%)	0
2012	71	33(56.0%)	3(5.1%)	31(52.5%)	0	0	29(93.5%)	0	0
2013	46	31(67.4%)	4(8.7%)	11(24.0%)	0	0	11(100%)	0	0
2014	53	26(61.9%)	3(7.1%)	24(57.1%)	0	8(33.3%)	16(66.6%)	0	0
2015	1,603	121(7.5%)	8(0.5%)	1,474(86.3%)	0	1,147(82.9%)	236(17.0%)	1(0.07%)	0
Total	10,051	1,215(12.1%)	338(3.4%)	8,251(82.1%)	4,742(57.5%)	1,155(14.0%)	1,511(18.3%)	649(7.90%)	9(0.1%)

Epidemiological data are from the Direction of Surveillance and Response to Epidemics (DSRE), Ministry of Public Health (MoPH); Microbiological Data from CERMES Abbreviations: Hi, *Haemophilus influenzae*, Nm, *Neisseria meningitis*; NmA, *Neisseria meningitidis* group A; NmC, *Neisseria meningitidis* group C; NmW, *Neisseria meningitidis* group W; NmX, *Neisseria meningitidis* group X; NmY, *Neisseria meningitidis* group Y; Spn,*Streptococcus pneumonia*.

## Discussion

From 2003 to 2015, meningitis outbreaks recorded in Niger have been dynamic with number of reported cases and variation of etiological Nm serogroup. However the number of reported cases has declined from 2010. This decrease may be due to the introduction of the vaccine against NmA, but also due to improvement of the surveillance system. *N. meningitidis* is the most important pathogen implicated in the occurrence of most recorded BM. NmA was responsible for the major epidemic waves from 2003 till 2009. In 2011, NmA has almost disappeared (only 5 cases reported across the country) with the introduction of the polysaccharide-tetanus toxoid conjugate vaccine PsA-TT in 2010. A total population number of 10,575,365 out of 10,870,817 target population aged between 1-to 29-years old were immunized with PsA-TT from 2010 through 2012, making coverage 97% [5]. While the NmA was disappearing progressively in 2010 the resurgence of NmW and NmC became evident and significantly pronounced in 2015. This situation has encouraged most investigators to hypothesize whether serogroup replacement has occurred and is somehow related to vaccination against NmA. Recognized as very severe, case-fatality rates for pneumococcal meningitis were reported to range from 27% to 80% globally [6]. *S. pneumonia* was found as the second main pathogen responsible for BM in Niger after *N. meningitidis*.

In 2014, Pneumococcal Conjugate Vaccine (PCV) was introduced in routine immunization program across the country. Although the number of reported cases of *S. pneumonia* meningitis was relatively poor (n = 26) in 2014, it would be premature to appreciate the success of the introduction of PCV. Watt JP et al (2009) reported a global estimates of the burden of disease caused *H. influenzae* type b in children younger than 5 years, 8.13 million cases with 371,000 deaths [7]. In settings without routine vaccination that target *H. influenzae* type b, HibCV (*H. influenzae* b Conjugate Vaccine) is expected to prevent 42% of all bacterial meningitis cases. The introduction of conjugate H. influenzae vaccine in infant immunization programs has led to drastic reduction or even elimination of Hib meningitis from several developed countries [8]. G. Campagne et al (1999) reported an estimate for the incidence of meningitis due to *H. influenzae* of 195/100,000 children less than one year of age and 50/100,000 children less than five years of age in a review of data regarding BM in Niamey, Niger [9]. The introduction of Hib vaccine presented as DTP-HepB-Hib in national immunization programs in 2008, has considerably contributed to an overall decline in reported *H. influenzae* meningitis [10, 11]. Epidemiological monitoring of BM through molecular characterization of implicated strains is very indispensable for high-level discriminations between isolates. The occurrence of 185(2.2%) cases of Nm meningitis of undetermined serogroup (non-typable) reflects the need for improvement and or advancement of laboratory molecular methods and practices in Niger. Although a panel of PCR procedures do exist for the identification of six important serogroups of Nm (A, C, W, X, Y and B) implicated in the development of meningitis in Niger, advanced molecular methods should be considered.

## Conclusion

The critical review and analysis of the national surveillance data for BM from 2003 to 2015, demonstrates the rank order of implicated pathogens, the success of PsA-TT vaccine against NmA, and overall, the unpredictability of the epidemiological characteristics of meningococcal meningitis in Niger. Since our research center is a referral one for eight regions of the country, we believe that our data together with that from the DSRE/MoPH may be used as surrogate makers of what is happening in the country. Given the burden associated with bacterial meningitis and the success of PsA-TT vaccine against NmA, we believed that most efforts should be focused in strengthening epidemiological and microbiological surveillance system and on preventing cases through routine vaccination programs across the country. This study highlight the need to monitor the evolution and distribution of Nm of other serogroups, the need in the medium term of the availability of a new, safe and affordable multivalent conjugate vaccine to respond to non-NmA meningococcal epidemics in future epidemic response and the need for the broadening of coverage for PCV13 vaccination programs against *S. pneumonia*.

### What is known about this topic

- Meningitis is a mortal serious public health disease in Niger;
- The main bacterial pathogens responsible for meningitis are known;
- The prevention of the disease through mass vaccination in most vulnerable areas across the country was found very successful.

### What this study adds

- Demonstrates the rank order of implicated bacteria pathogens responsible of meningitis epidemics in Niger from 2003 to 2015;
- Demonstrates the success of vaccinations strategies against bacterial meningitis;
- Demonstrates the unpredictability of the epidemiological characteristics of meningococcal meningitis in Niger.

## Competing interests

The authors declare no competing interest.
